# MLWAN: Multi-Scale Learning Wavelet Attention Module Network for Image Super Resolution

**DOI:** 10.3390/s22239110

**Published:** 2022-11-24

**Authors:** Jian Ma, Xiyu Han, Xiaoyin Zhang, Zhipeng Li

**Affiliations:** 1 School of Computer Science, Fudan University, Shanghai 200433, China; 2 School of Internet, Anhui University, Hefei 230039, China

**Keywords:** multi-scale image super resolution, channel-spatial attention mechanism, channel attention recurrent module, inverse discrete wavelet transform

## Abstract

Image super resolution (SR) is an important image processing technique in computer vision to improve the resolution of images and videos. In recent years, deep convolutional neural network (CNN) has made significant progress in the field of image SR; however, the existing CNN-based SR methods cannot fully search for background information in the measurement of feature extraction. In addition, in most cases, different scale factors of image SR are assumed to be different assignments and completed by training different models, which does not meet the actual application requirements. To solve these problems, we propose a multi-scale learning wavelet attention network (MLWAN) model for image SR. Specifically, the proposed model consists of three parts. In the first part, low-level features are extracted from the input image through two convolutional layers, and then a new channel-spatial attention mechanism (CSAM) block is concatenated. In the second part, CNN is used to predict the highest-level low-frequency wavelet coefficients, and the third part uses recursive neural networks (RNN) with different scales to predict the wavelet coefficients of the remaining subbands. In order to further achieve lightweight, an effective channel attention recurrent module (ECARM) is proposed to reduce network parameters. Finally, the inverse discrete wavelet transform (IDWT) is used to reconstruct HR image. Experimental results on public large-scale datasets demonstrate the superiority of the proposed model in terms of quantitative indicators and visual effects.

## 1. Introduction

Image super resolution (SR) reconstruction technology refers to the process of restoring a given low-resolution (LR) image into a corresponding high-resolution (HR) image by a specific algorithm. It is designed to overcome or compensate the problems of blurred image, low quality, and insignificant region of interest caused by the limitations of image acquisition system or acquisition environment. At present, many sophisticated visual applications (e.g., satellite and aerial imaging [1], medical imaging [2,3], and security and surveillance imaging [4,5]) can benefit from high-quality reconstructed HR images when SR techniques are used as a preprocessing step. How can we extract valuable information from the various kinds of LR images is the key to solving SR problems. Since the texture structure of an image has infinite solutions in the process of image SR reconstruction, it is a typical ill-posed problem.

To solve the SR problem, there are usually both hardware and software approaches. On the hardware side, this can be achieved by reducing the size of individual sensors; however, that would raise prices exponentially. The software method to achieve the image SR reconstruction has basically no cost; therefore, in earlier research, the mainstream algorithms of image SR are mainly divided into three categories: interpolation-based methods, reconstruction-based methods, and learning-based methods. More specifically, interpolation-based SR methods, such as bicubic interpolation [6] and Lanczos resampling [7], are fast and simple, but lack accuracy. Reconstruction-based SR methods [8,9,10,11] usually use complex prior knowledge to limit the space of possible solutions, and have the advantage of generating flexible and clear details; however, the performance of many reconstruction-based methods deteriorates rapidly as scaling factors increase, and these methods are often time consuming. Learning-based SR methods, also known as instance-based methods, have gained attention due to their fast computation and excellent performance. These methods typically utilize machine learning algorithms to analyze the statistical relationship between LR and its corresponding HR counterpart from substantive training samples. For instance, Freeman et al. first adopted Markov Ranomom Field (MRF) [12] to synthesize visually pleasing image textures with rich real-world images. Chang et al. [13] proposed a neighborhood embedding method for image SR, which used similar local geometry between LR and HR to recover HR image patches. Motivated by sparse signal recovery theory [14], researchers have applied sparse coding methods [15,16] to SR problems. In addition, random forests [17] have also been used to improve reconstruction performance.

Recently, with the remarkable performance of deep learning (DL) technology in the field of computer vision, a variety kinds of DL-based image SR methods have been applied to solve image SR problems. For instance, ranging from the early convolutional neural networks (CNNs)-based methods (e.g., SRCNN [18,19]) to recent promising image SR models using generative adversarial nets (GAN) [20] (e.g., SRGAN [21]). Generally, image SR algorithms using DL technology differ from each other can be categorized into three main areas: different types of network architectures [22,23,24], different types of loss functions [25,26], different types of learning principles and strategies [25,27], etc. All of the DL-based SR approaches have demonstrated great superiority to reconstruction-based and other learning-based methods. However, most early DL-based image SR methods techniques typically use learnable up-sampling layers, such as deconvolution and subpixel convolution [28], to obtain the desired size. Unfortunately, when the deep network can only optimize one scale factor, the upsampling of HR images can only be achieved at a specific scale. As a result, more calculations and memory regions are needed for model training and storage when a multi-scale SR algorithm is being implemented. In addition, in order to improve the performance of SR, additional network parameters and long inference time have become a prevalent issue. To solve this issue, some researchers have proposed two network shrinkage techniques while maintaining performance. One is to carefully design a condensed architecture [29,30], which will be effective but requires a high level of expertise. The other is to use parameter sharing algorithms [31,32], such as recursive/cyclic learning, but this class of methods uses sequential inference methods for each recursive/recursive block, which makes the inference time longer. In addition, many CNN-based SR methods ignore the feature connection in the middle layer, which leads to the texture details of LR images tend to be smooth in SR output. Although the results achieved by utilizing channel attention [33,34] maintain some detailed information, the channel attention-based techniques fail to preserve useful textures and restore natural features. Because they handle the feature maps at various levels identically, which results in certain detail sections being lost in the reconstructed image; therefore, SR research still has challenges in creating texture details.

**Figure 1:**
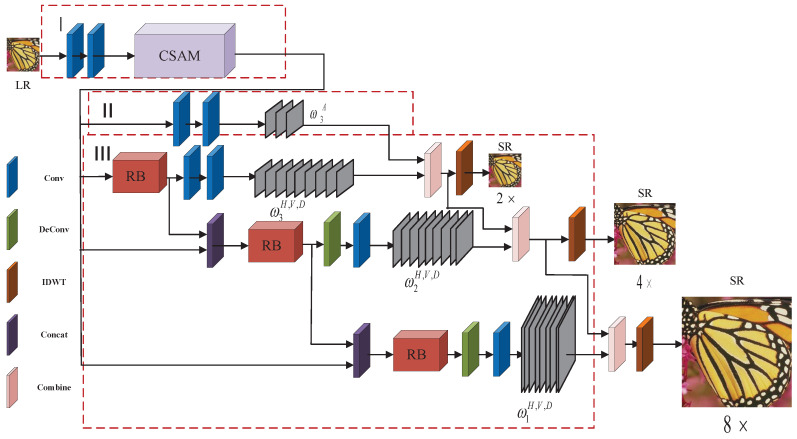
The overall framework of the proposed model (MLWAN).

Based on the above analysis, the current methods do not form a balance in pursuit of model inference time, parameters, and performance. Motivated by these facts, we propose a multi-scale learning wavelet attention network (MLWAN) model to achieve fast, accurate, and lightweight SR tasks. The overall architecture of the proposed MLWAN model is shown in Figure 1. As depicted in Figure 1, the proposed model can utilize more context-specific information and achieve multi-scale SR through a single-scale SR model. Compared with the previous wavelet correlation methods [35,36,37], our network uses the original LR image as direct input at different levels and predicts the wavelet coefficients of the target image. To be specific, the whole network is principally divided into three portions. In the first portion, two convolutional layers and a new channel-spatial attention mechanism (CSAM) are used to extract low-level features from the input LR images. In the second portion, it is composed of CNN branches to predict the highest-level low-frequency wavelet coefficients. The third part is an RNN branch, the remaining subband coefficients are predicted by the RNN component. In addition, an efficient channel attention recurrent module (ECARM) is proposed to compose the RNN branch. It reduces the total size of the network parameters by using the approach of sharing parameters. Moreover, different from anterior recurrent neural networks (RNN)-based approaches [31,32,38], the number of real recurrences in this work is determined by the scale factor, and every recurrence is responsible for predicting a certain level of subband coefficients. Based on the predicted wavelet coefficients of each branch, the HR image is reconstructed by inverse discrete wavelet transform (IDWT). Note that a preliminary version of this paper is published in [39], which only learn simple wavelets and channel attention network for image SR. This presented work adds some new insights to the preliminary version so that the proposed MLWAN model has better robustness and universality.

In general, the mainly contributions of this work are as follows:

(1) A multi-scale learning wavelets attention network (MLWAN) is proposed to complete multi-scale SR task in a fast and lightweight way. The network predicts the wavelet coefficient of the source image, and inverts the predicted wavelet coefficients to obtain the final HR image.
(2) A novel channel-spatial attention mechanism (CSAM) block is proposed to learn the channel and spatial correlation of each layer’s features. Due to it including responses from all feature mapping dimensions, we use it in our network to extract low-level features of LR image more completely.
(3) As the basic unit in recurrent block (RB), an efficient channel attention recurrent module (ECARM) is proposed for reducing the network parameters. Experimental results show that the proposed MLWAN network achieves a good balance in model inference time, parameters, and performance, and outperforms most of the existing SR methods.

## 2. Related Works

Because of the high approximating capacity and hierarchical property of an artificial neural network (ANN), most modern image SR models are based on DL technology. In this section, we analyze the current DL-based models in the SR field from three aspects: wavelet-related SR, RNN-based SR, and attention mechanisms.

### 2.1. Wavelet-Related SR

As is known to all, wavelet transform is an efficient image representation method, which decomposes image signals into high frequency subbands representing texture details and low frequency subbands containing global topological information. Bae et al. [40] first combined the wavelet transform with deep-learning-based SR model, took the subband of the interpolated LR wavelet as the input, and predicted the residual value of the corresponding HR subband. Wavelet transform and inverse wavelet transform are used to decompose LR input and reconstruct HR output, respectively. Later, with the in-depth study of deep learning, various SR algorithms combining deep learning and wavelet transform have been proposed. Guo et al. [35] presented a deep wavelet super-resolution (DWSR) model by using a deep CNN. Liu et al. [36] developed a multi-level wavelet-CNN (MWCNN) model by using the inverse wavelet transform and the discrete wavelet transform in the process of up-sampling and down-sampling. Additionally, Xue et al. [37] constructed a wavelet-based residual attention network by stacking several multi-kernel convolutional layers that are applied by the attention block (WRAN). Ji et al. [41] predicted the missing portion of the wavelet coefficient using the multi-frame information in the wavelet domain. Anbarjafari et al. [42] proposed a novel SR metric based on interpolation of wavelet domain high frequency subbands and the spatial domain input image. Zhang et al. [43] proposed a lightweight and fast network (MSWSR) to implement multi-scale SR simultaneously by learning multi-level wavelet coefficients of the target image. Huang et al. [44] presented a wavelet-based CNN approach for face images SR. In summary, most of these wavelet correlated SR networks input a wavelet coefficient layer from the bicubic upsampled LR image and output a wavelet coefficient layer from the desired image, which can be considered as refining the wavelet coefficients of the upsampled LR image to the wavelet coefficients of the matched HR image. Most importantly, although these methods provide a high sampling scale, they do not take full advantage of wavelet transform in multiresolution decomposition. To perform multi-scale SR model, in this study, the original LR image is directly used as the input image to establish a simple network to predict the wavelet coefficients of the target image.

### 2.2. RNN-Based SR

The RNN is a recurrent fully connected neural network model inspired by the spiking behavior of biophysical neurons, which has achieved remarkable results in many tasks of natural language processing (NLP). On one hand, the feedback mechanism of RNN allows the model to use the current output to change the previous state. On the other hand, RNN is similar to ordinary artificial neural networks, with a tree-like hierarchical system, in which network nodes recursively input data in the order of connection. Generally, it is used for machine learning problems related to structural relationships, with flexible topology and weight sharing. In addition, RNN and recurrent neural networks are often used to reduce model parameters because they adopt the method of parameter sharing [31,32,38]. For instance, Kim et al. [45] proposed a deeply-recursive convolutional network (DRCN) to improve performance and reduce model parameters. Tai et al. [32] proposed a deep recursive residual network (DRRN) model by using a similar recursive mechanism and residual blocks [46]. Li et al. [31] proposed an SRFBN model including four cyclic inference units, each of which has a complex structure, resulting in a long inference time. Obviously, by recursively inferring recursive units, these methods greatly reduce the total number of network parameters. However, because so much recursion is involved, it can lead to long inference time. To perform multi-scale SR in a lightweight and fast way, in this paper, RNN algorithm is used to achieve the balance between the total network parameters and reconstruction performance. Meanwhile, we also attempt to shorten the inference time.

### 2.3. Attention Mechanism

Attention mechanism is derived from human visual attention mechanism, which can be regarded as an application of bionics. A brain signal processing system specific to human eyesight is called the visual attention mechanism, which has played a key role in various fields of computer vision (e.g., image recognition, capture, and restoration). The power to differentiate is given to models by the attention, which makes it highly fashionable. For instance, in speech recognition and machine translation applications, each word in the sentence is given a different weight, which increases the learning flexibility (softness) of the neural network block. In addition, attention can be used as an alignment relation to explain the relationship between output and input sentences and the knowledge acquired by the interpretation network. It gives us a window into the black box of deep learning. In other words, they assist the network in concentrating on crucial information while dismissing unimportant information. At present, the research object of image SR algorithm has shifted from traditional CNN to attention-based DL. For instance, Wang et al. [47] presented a residual attention network based on the truck-and-mask attention mechanism. Hu et al. [48] proposed a SE-Net model by using the channel attention learning mechanism. Zhang et al. [33] developed a residual channel attention network (RCAN) by using an extremely deep network with channel attention of SR. Zhu et al. [49] utilized U-shaped formation and the residual channel attention block to achieve excellent image SR performance. Woo et al. [50] used spatial attention (SA) and channel attention (CA) blocks to take advantage of the inter-spatial and inter-channel relationship of feature maps. Based on mentioned-above, clearly, the construction of complex attention module can improve the performance of image SR. Based on our previous work [39], in this study, we present a novel CSAM block to study the interdependencies between pixels and channels.

## 3. Method

**Figure 2:**
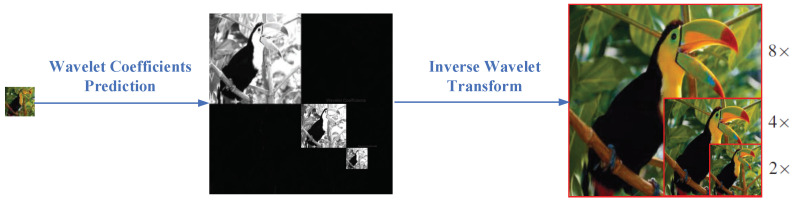
The main idea of the proposed (2, 4, 8)× MLWAN.

In this section, we describe the proposed MLWAN and the adopted loss function in details. As shown in Figure 2, the main idea of this study is to make use of the characteristics of wavelet transform, that is, one level of wavelet coefficients can generate a 2× image by inverse wavelet transform. Taking the three-level wavelet as an example, the wavelet coefficients of the target HR image are predicted from the corresponding LR image. Then, the target HR images with 2×, 4×, and 8× scales are reconstructed by multi-level inverse wavelet transform, respectively.

### 3.1. Overview of the Proposed Model

As discussed above, some DL-based SR tasks (such as 2×, 4×, and 8×) can effectively deal with single-scale SR, while multi-scale SR is still difficult. In this paper, we propose a novel MLWAN model for multi-scale SR. The network framework is illustrated in Figure 1. It is divided into three main parts. In the first part, we use two convolutional layers to extract low-level features from the input image. Then, a CSAM block is concatenated to extract the underlying features representing the intra-channel and inter-channel information in a continuous channel. Furthermore, we use one CNN part and one RNN part to predict the wavelet coefficients of the target image. In the CNN part, two successive convolutional layers process the extracted low-level features to predict the wavelet coefficients $\omega_{3}^{A}$. In addition, in order to reduce the total number of model parameters, an efficient channel attention recurrent module (ECARM) is introduced in the RNN part, which adopts the method of sharing all parameters. The ECARM module takes the low-level features as the input and cooperates with two following convolutional layers to predict the three remaining third-level wavelet coefficients (i.e., $\omega_{3}^{H}$, $\omega_{3}^{V}$, and $\omega_{3}^{D}$). Consequently, 2D IDWT of the third-level wavelet coefficients is applied to calculate the $O_{2\times }$. Subsequently, the ECARM with the same weights takes the concatenation of its previous output and the low level feature as input to generate discriminative features. Then, a 2× deconvolutional layer and a convolutional layer are utilized to predict the second-level wavelet coefficients (i.e., $\omega_{2}^{H}$, $\omega_{2}^{V}$, and $\omega_{2}^{D}$) from the generated features; therefore, the second-level wavelet coefficients are inversely transformed into the $O_{4\times }$ by the corresponding IDWT. Likewise, the same RB propagates one more time followed by a 4× deconvolutional layer and a convolutional layer to predict the first-level wavelet coefficients (i.e., $\omega_{1}^{H}$, $\omega_{1}^{V}$, and $\omega_{1}^{D}$). Ultimately, the $O_{8\times }$ is reconstructed. Note that we adopt the db1 wavelet function as a wavelet filter, and all of the RBs share the same weights.

As depicted in Figure 1, the proposed MLWAN can reconstruct 2×, 4×, and 8× SR images using a single network, while the magnification of SR images has grown exponentially with the recurrent times of RB. Thus, at the inference step, SR results with the desired scale can be achieved by considering the scale factor to flexibly control the recurrent times of RB. In theory, since the RNN branch can involve an arbitrary number of recurrences, the proposed MLWAN network is scalable and has the ability to deal with multi-scale SR tasks via a single network if the scale factor is a power of 2. Moreover, benefiting from the weight sharing strategy of RBs, fewer additional parameters are involved when extending the RNN part to obtain more SR scales.

### 3.2. The Channel-Spatial Attention Mechanism (CSAM) Module

**Figure 3:**
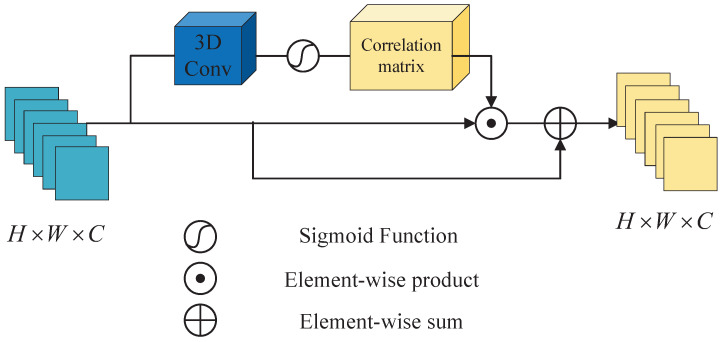
The overview of the proposed channel-spatial attention module (CSAM).

Attention mechanism has made important breakthroughs in the field of image processing and NLP in recent years, which has been proved to be beneficial to improve the performance of the model. The essence of attention mechanism is to locate the information of interest and suppress useless information. The results are usually displayed in the form of probability maps or probabilistic feature vectors. According to different application scenarios, it is mainly divided into spatial attention model, channel attention model, and channel-spatial mixed attention model [33,34,50,51,52]. As is well known, channel attention focuses on what kind of features are meaningful, while spatial attention focuses on where features are meaningful. In [50], the two modules, channel attention and spatial attention, can be combined in parallel or sequentially, and the authors found that combining them sequentially and putting channel attention first leads to better results. Inspired by these findings, we propose a new channel-spatial attention mechanism (CSAM) that incorporates the response from every dimension of feature maps. To be specific, the overview of the CSAM is shown in Figure 3. The input feature $F_{N}$ is fed into a 3D convolution layer [53] to build an attention map by collecting joint spatial and channel characteristics, granted the output layer features maps $F_{N}\in R^{H\times W\times C}$. We then apply 3D convolutions, using a kernel size of 3, 3×3×3, stride of 1 and padding of 1 on each of the 3 channels separately to generate three sets of channel-spatial attention mappings $W_{csa}$.

Furthermore, we use element-wise product operation on the input feature $F_{N}$ and the attention map $W_{csa}$. Finally, the weighted features $F_{CS}$ are defined by

(1) $$F_{CS}=\beta \sigma \left(W_{csa}\right)⊙F_{N}+F_{N}$$

where β is a scale factor, ⊙ is the element-wise product, σ(·) is the sigmoid function. As a result, $F_{CS}$ is the weighted sum of every spatial-channel position feature along with the primary feature. In general, the proposed CSAM explicitly models spatial feature inter-dependencies and channel-wise to adaptively learn the intra-channel and inter-channel feature responses.

### 3.3. The Efficient Channel Attention Recurrent Module (ECARM)

**Figure 4:**
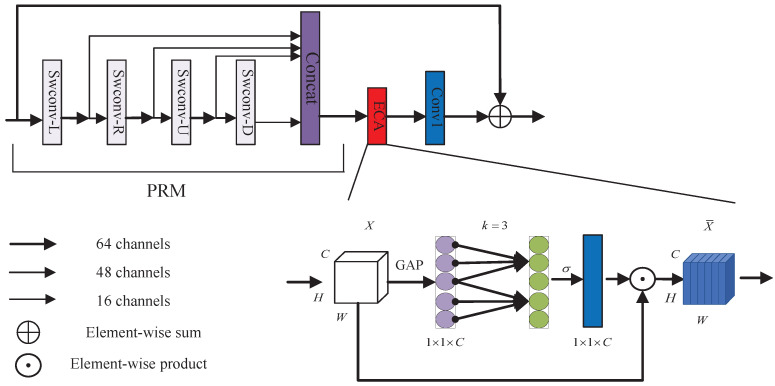
Diagram of our efficient channel attention recurrent module (ECARM).

Recently, channel attention mechanisms have been shown to have great potential in improving the performance of deep CNNs; however, most existing approaches focus on developing more complex attention modules to achieve better performance, which inevitably increases the complexity of the model [48]. In order to overcome the contradiction between performance and complexity tradeoff, we propose an effective channel attention recurrent module (ECARM), which contains only a few parameters and brings significant performance improvement. As depicted in Figure 4, the proposed ECARM is composed by progressive refinement module (PRM), efficient channel attention (ECA) module [54], and one 1×1 convolution layer. Note that our ECARM as a whole uses residual connections [46]. As shown in the gray box in Figure 4, PRM uses a convolution layer with the size of 3×3 to extract the input features of several successive distillation stages. Then, in each step, we use a channel split operation on the features from the previous stage to create two partial features. One part is saved and the remaining part is used in the following computation unit. The portion that was kept might be thought of as the refined features. Specifically, we utilize a band matrix $W_{k}$ to learn channel attention, and $W_{k}$ can be defined by

(2) $$W_{k}=\left[\begin{array}{cccccccc} w^{1,1} & \cdots & w^{1,k} & 0 & 0 & \cdots & \cdots & 0\\ 0 & w^{2,2} & \cdots & w^{2,k+1} & 0 & \cdots & \cdots & 0\\ \vdots & \vdots & \vdots & \vdots & \ddots & \vdots & \vdots & \vdots \\ 0 & \cdots & 0 & 0 & \cdots & w^{C,C-k+1} & \cdots & w^{C,C} \end{array}\right]$$

where $W_{k}$ includes k×C parameters. The weight of $y_{i}$ is calculated by only considering interaction between $y_{i}$ and its *k* neighbors, as follows:

(3) $$\omega_{i}=\sigma \left(\sum_{j=1}^{k}\omega_{i}^{j}y_{i}^{j}\right),y_{i}^{j}\in \Omega_{i}^{k}$$

where σ is a sigmoid function. The $\Omega_{i}^{k}$ is the set of *k* adjacent channel of $y_{i}$. Then, a more efficient approach is to make all channels share the same learning parameters, as follows:

(4) $$\omega_{i}=\sigma \left(\sum_{j=1}^{k}\omega^{j}y_{i}^{j}\right),y_{i}^{j}\in \Omega_{i}^{k}$$

Note that we can easily implement this strategy with a fast 1D convolution of kernel size *k* as follows:

(5) $$\omega =\sigma \left(C1D_{k}\left(y\right)\right)$$

where the C1D refers to 1D convolution. Here, the method in Equation (5) is called by ECA module, which only involves *k* parameters. In a word, it ensures both efficiency and effectiveness by appropriately capturing local cross-channel interaction.

### 3.4. Loss Functions

In this section, the pairwise difference between a reconstructed HR image and its corresponding ground truth (GT) is measured at two stages and two different domains. The losses take into account several factors, including the overall pixel distance between images in the spatial domain, as well as frequency and texture differences in the wavelet domain. The total loss is formed by linearly combination of loss components, as follows:

(6) $$Loss_{\mathrm{total}\;}=\alpha Loss_{\mathrm{spatial}\;}+\beta Loss_{\mathrm{wavelet}\;}$$

Spatial Loss $Loss_{\mathrm{spatial}}$: After each IDWT, the mean absolute error (MAE) between the reconstruction results of different scale factors ($O_{2\times }$, $O_{4\times }$, and $O_{8\times }$) and the corresponding GTs is calculated to minimize the pixel level difference in the spatial domain, which is formulated as:

(7) $$Loss_{\mathrm{spatial}\;}={∥O_{2\times }-bic_{4↓}\left(G\right)∥}_{1}+{∥O_{4x}-bic_{2↓}\left(G\right)∥}_{1}+{∥O_{8x}-G∥}_{1}$$

where *G* indicates the corresponding GT of $O_{8\times }$, and $bic_{s↓}\left(\cdot \right)$ is the function for bicubic downsampling with scale factors.

Wavelet Loss $L_{\mathrm{wavelet}}$: Unlike many other SR methods [27,31,33] that only introduce losses between output and ground truth at the spatial domain, we adopt a wavelet loss at the wavelet domain, to better constrain the distance between predicted wavelet coefficients and target wavelet coefficients before the IDWT. In addition, we use MAE to calculate the wavelet loss between the predicted wavelet coefficient (ω) and its corresponding GT to help generate more high-frequency and detailed textures, which can be expressed as follows:

(8) $$Loss_{\mathrm{wavelet}\;}={∥\omega -dwt_{n}\left(G\right)∥}_{1}$$

where $dwt_{n}\left(\cdot \right)$ is the mapping function for *n*-level 2D discrete wavelet transform (DWT).

## 4. Experiments

In this section, we first introduce the datasets and evaluation metrics used in our experiments. Then, the implementation details of the proposed model are discussed. On this basis, the effectiveness of the proposed MLWAN is compared quantitatively and qualitatively. Finally, the results of ablation experiments are presented.

### 4.1. Metrics and Datasets

DIV2K [55] is a popular image SR dataset that includes 1000 high-quality images of natural RGB images. In our experiments, the first 900 images of DIV2K are selected as the training data. Meanwhile, five standard benchmark datasets: Set5 [56], Set14 [16], B100 [57], Urban100 [58], and Manga109 [59] are used for evaluation. SR results are evaluated by two quantitative measures. Specifically, the peak signal-to-noise ratio (PSNR) and structural similarity index (SSIM) [60] are used to evaluate the quality of reconstructed HR images. For a fair comparison, the PSNR and SSIM are only calculated on the luminance (Y) channel as previous works do. Note that the Matlab functions with bicubic interpolation are used to downsample high-quality images to generate LR images of training data.

### 4.2. Implementation Details

The proposed MLWAN in our experiments is trained on (2, 4, 8)× SR for 4000 epochs. In every iteration, we randomly crop 32 patches with the size of 40×40 as the inputs. For dynamic data augmentation, we use random flips (horizontal and vertical) and a 90-degree rotation before feeding the data into the network. The network parameters are initialized according to [61] and optimized by Adam [62] with the learning rate of $4\times 10^{-5}$. Both α and β in Equation (6) are set to 1.0. Our MLWAN training codes are implemented with the PyTorch Library [63] run on the NVIDIA Quadro RTX 6000 GPU.

### 4.3. Effectiveness Analysis

In order to verify the effectiveness and robustness of the proposed MLWAN model, we choose nine representative state-of-the-art SR methods for comparison. These methods can be grossly divided into two groups: one includes six single-scale SR methods: Bicubic [6], SRCNN [18], FSRCNN [64], VDSR [22], and DRCN [45]; the other contains three multi-scale SR methods, including MemNet [38], MSSR [65], LapSRN [23], and MSWSR [43].

For network parameters, we consider convolutional, deconvolutional, and fully connected layers when calculating the parameters of the entire network. The parameter of IDWT is not included because its quantity is not obvious, specifically 0.008 K. The number of parameters for a convolutional layer can be defined by

(9) $$\;P\;=C_{\mathrm{in}\;}\times C_{\mathrm{out}\;}\times k_{h}\times k_{\omega }+b$$

where $C_{\mathrm{in}}$ and $C_{\mathrm{out}}$ are the numbers of input and output channels in the convolutional layer, respectively. Note that ($k_{h}$, $k_{\omega }$) is the kernel size. *b* is the number of bias, which is equal to $C_{\mathrm{out}}$ in terms of bias 0 when no bias is used. The size of the bias controls how easy it is to activate the sensor. When we use bias, it equals $C_{\mathrm{out}}$. In addition, for parameters of the deconvolution layer, the parameters are calculated in the same way as those of the convolutional layer.

The number of parameters for a fully connected layer can be calculated by

(10) $$\;P\;=D_{\mathrm{in}\;}\times D_{\mathrm{out}\;}+b$$

where $D_{\mathrm{in}}$ and $D_{\mathrm{out}}$ are the numbers of input and output dimensions in the fully connected layer, respectively. *b* is the number of bias, is equal to $C_{\mathrm{out}}$ in terms of bias 0 when no bias is used. Note that In our experiments, all the numbers of network parameters are calculated based on Equations (9) and (10).

For inference time, in our experiments, all of the network inference time are measured on the B100 dataset that contains 100 images. The official implementation of the comparison method is based on different deep learning libraries, which utilize different computational backends, affecting the fairness of the inference time comparison. In order to reduce the measurement differences caused by different software or hardware, we only adopt the PyTorch Library on a NVIDIA Quadro RTX 6000 GPU when computing the network inference time.

In comparison experiments, we first compare the proposed MWLAN model with Bicubic [6], SRCNN [18], FSRCNN [64], VDSR [22], DRCN [45], and MemNet [38]. The overall performance results on Set5 [56], Set14 [16], B100 [57], Urban100 [58], and Manga109 [59] databases are tabulated in Table 1. As shown in Table 1, we can observe that the proposed MWLAN model has a great improvement in PSNR and SSIM contrast with Bicubic, SRCNN, and FSRCNN. Particularly, for the VDSR [48], the proposed model has higher PSNR and SSIM of 4× and 8× images. Moreover, compared with the 4× Set5 dataset, PSNR and SSIM are improved by 0.84 and 0.0127. Then, for the DRCN [45], the proposed method is not just higher in PSNR and SSIM than the former, but also less in parameters and inference time. For the MemNet [38], the inference time of the proposed network is much less than it. In conclusion, the performance of the proposed MWLAN model in terms of SSIM and PSNR are better than other comparison models. Furthermore, to verify the effectiveness of multi-scale SR via a single network, we compare the proposed MWLAN model with MSSR, LapSRN, and MSWSR, which are originally designed for multi-scale SR tasks in Table 2. As shown in Table 2, we can observe that although the inference time of LapSRN is very short, the proposed MWLAN model achieves better PSNR and SSIM values than the LapSRN. Especially, on the Manga109 dataset of 8× images, the proposed MWLAN method improves PSNR by 1.12 and SSIM by 0.0483. Obviously, the proposed MWLAN model achieves better PSNR/SSIM results than MSSR, LapSRN, and MSWSR methods. Accordingly, we can conclude that the proposed MWLAN model is powerful for performing multi-scale SR in a lightweight and fast way.

### 4.4. Ablation Study

#### 4.4.1. Quantitative Analysis

**Figure 5:**
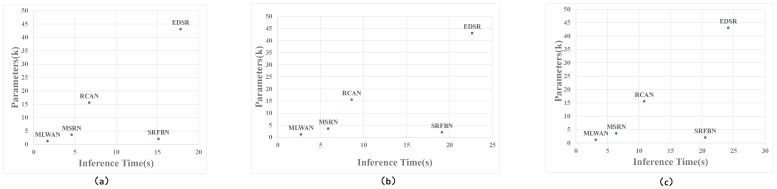
Comparisons on the number of model parameters and running time of the SR methods with different scale sizes. (**a**) The results for 2× images. (**b**) The results for 4× images. (**c**) The results for 8× images.

In addition, we also make the comparisons on the number of model parameters and running time of the SR methods with different scale sizes. The results are shown in Figure 5. As can be seen from Figure 5, for EDSR [27] and RCAN [33], their PSNR and SSIM values are higher than the proposed model, but their parameters and inference time are much larger than the proposed method. Recurrent learning is used in SRFBN [31] to lower the all number of model parameter, however the suggested model’s parameters and inference time are also higher. Although these methods are higher than the proposed methods in PSNR and SSIM values, they are far lower than the proposed methods in the network parameters and inference time. Through quantitative comparison with the above methods, we find that the proposed method realizes a better balance in three fields: reconstruction performance, model parameter, and inference time; therefore, the proposed method can be embedded into the real-time image processing system for image SR applications.

#### 4.4.2. Visual Effect Analysis

**Figure 6:**
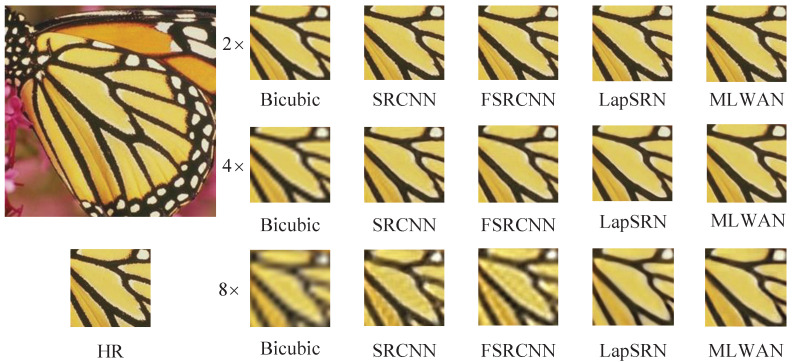
Visual comparisons of (2, 4, 8)× SR with different SR advances on Set5 butterfly.

**Figure 7:**
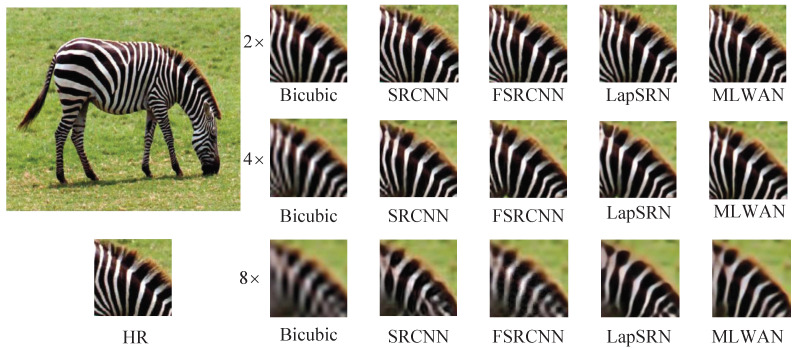
Visual comparisons of (2, 4, 8)× SR with different SR advances on Set14 zebra.

**Figure 8:**
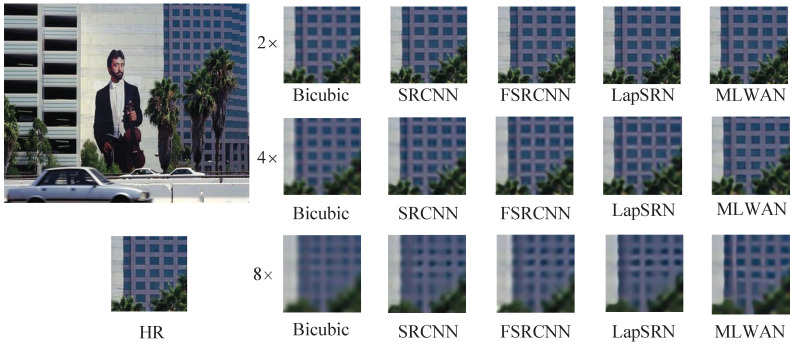
Visual comparisons of (2, 4, 8)× SR with different SR advances on B100 119082.

**Figure 9:**
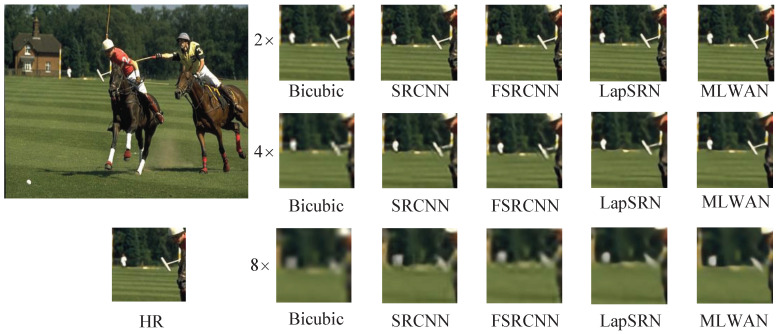
Visual comparisons of (2, 4, 8)× SR with different SR advances on B100 361010.

In this section, we visually compare the proposed method with some representative SR methods. Specifically, we compare the proposed MLWAN with Bicubic [6], SRCNN [18], FSRCNN [64], and LapSRN [23], and perform SR reconstruction of 2×, 4× and 8× images on standard datasets. The experimental results are shown in Figure 6, Figure 7, Figure 8 and Figure 9. Note that the image of Figure 6 is selected in Set5 [56], the image of Figure 7 is selected in Set14 [16], and the images of Figure 8 and Figure 9 are selected in B100 [57]. Obviously, it can be seen that the proposed method has better and clearer texture and reconstruction effect than the previous four methods. Especially, when 4× and 8× scales are used, the comparison of the results suggests that the proposed algorithm outperforms the other SR methods. In conclusion, based on subjective image quality assessment (IQA), we can find that the subjective image quality perception of the proposed model significantly outperforms the current representative SR algorithms.

#### 4.4.3. The Effectiveness of CSAM

The extracted features by the CSAM block may have a great influence on the performance of the whole network. Based on such consideration, we design two groups of experiments. The first experiment is MLWAN without CSAM block that is named by MLWAN (without CSAM). The second experiment is MLWAN with CSAM block. We test the two trained networks on five standard benchmark datasets for 2×, 4×, and 8× in terms of SSIM and PSNR. The experimental results are shown in Table 3. As can be seen in Table 3, we can observe that the proposed model with CSAM block has evidently higher PSNR and SSIM values than the model without CSAM block; therefore, the CSAM block plays a key role in enhancing the performance of the proposed model. In general, the proposed CSAM block can efficiently extract image structure and texture information for image SR.

#### 4.4.4. The Influence of ECA on RB

To verify the availability of the ECA module, we trained a new model without the ECA module from RB block in the proposed model, and compared it with the initial MLWAN. The results are shown in Table 4. We test the two models on five standard benchmark datasets for 2×, 4×, and 8× in terms of SSIM and PSNR. The PSNR and SSIM values obtained show that the proposed MLWAN without ECA are significantly lower than the initial MLWAN; therefore, we can conclude that overall performance can be enhanced by embedding the ECA in the proposed MLWAN model.

#### 4.4.5. Comparison with Our Previous Work

In our previous work [39], we proposed a single scale model for 4× image SR, called LWCAN. Please refer to the literature [39] for details. In this section, in order to demonstrate the universality and robustness of the proposed algorithm, we compare the proposed MLWAN model with LWCAN in terms of PSNR and SSIM. Experimental results are shown in Table 5. Since the previous model is designed for 4× SR, we can only compare the 4× scale in this study. As can be seen in Table 5, clearly, it can be seen that the proposed model is better than the previous model [39] in terms of PSNR and SSIM. In conclusion, the proposed model has better robustness and versatility than the LWCAN metric.

## 5. Conclusions and Future Work

In this paper, we propose a multi-scale learning wavelet attention network (MLWAN) to achieve accurate, fast, and lightweight image super resolution. The novelty of the proposed MLWAN model is that we use CSAM blocks in feature extraction and ECARM in RNN branching to improve network performance. Particularly, the proposed MLWAN model achieves a balance between network reconstruction performance, inference time, and parameter number. Experimental results show that the proposed model outperforms some representative SR methods. In the future work, we plan to extend the proposed approach to video super resolution.

## Figures and Tables

**Table 1:** Comparisons on the number of network parameters, inference time, and PSNR/SSIM of different single-scale SR methods.

Method	Scale	Params	Times	PSNR/SSIM				
				Set5	Set14	B100	Urban100	Manga109
Bicubic [6]	2×	-	-	33.66/0.9299	30.24/0.8688	29.56/0.8431	26.88/0.8403	30.80/0.9339
SRCNN [18]		8 K	-	36.66/0.9542	32.45/0.9067	31.36/0.8879	29.50/0.8946	35.60/0.9663
FSRCNN [64]		13 K	-	37.05/0.9560	32.66/0.9090	31.53/0.8920	29.88/0.9020	36.67/0.9710
VDSR [22]		666 K	1.80 s	37.53/0.9590	33.05/0.9130	31.90/0.8960	30.77/0.9140	37.22/0.9750
DRCN [45]		1775 K	33.38 s	37.63/0.9588	33.04/0.9118	31.85/0.8942	30.75/0.9133	37.55/0.9732
MemNet [38]		678 K	11 s	37.78/0.9597	33.28/0.9142	32.08/0.8978	31.31/0.9195	37.72/0.9740
MLWAN (ours)		1230 K	1.69 s	37.68/0.9589	**33.47/0.9227**	31.96/0.8974	**31.34/0.9309**	37.39/0.9733
Bicubic [6]	4×	-	-	28.42/0.8104	26.00/0.7027	25.96/0.6675	23.14/0.6577	24.89/0.7866
SRCNN [18]		8 K	-	30.48/0.8628	27.50/0.7513	26.90/0.7101	24.52/0.7221	27.58/0.8555
FSRCNN [64]		13 K	-	30.72/0.8660	27.61/0.7550	26.98/0.7150	24.62/0.7280	27.90/0.8610
VDSR [22]		666 K	2.26 s	31.35/0.8830	28.02/0.7680	27.29/0.7260	25.18/0.7540	28.83/0.8870
DRCN [45]		1775 K	41.54 s	31.53/0.8854	28.02/0.7670	27.23/0.7233	25.14/0.7510	28.93/0.8854
MemNet [38]		678 K	13.76 s	31.74/0.8893	28.26/0.7723	27.40/0.7281	25.50/0.7630	29.42/0.8942
MLWAN (ours)		1230 K	2.70 s	**32.19/0.8957**	**28.55/0.7797**	**27.67/0.7429**	**25.89/0.7779**	**30.16/0.9110**
Bicubic [6]	8×	-	-	24.40/0.6580	23.10/0.5660	23.67/0.5480	20.74/0.5160	21.47/0.6500
SRCNN [18]		8 K	-	25.33/0.6900	23.76/0.5910	24.13/0.5660	21.29/0.5440	22.46/0.6950
FSRCNN [64]		13 K	-	20.13/0.5520	19.75/0.4820	24.21/0.5680	21.32/0.5380	22.39/0.6730
VDSR [22]		666 K	2.40 s	25.93/0.7240	24.26/0.6140	24.49/0.5830	21.70/0.5710	23.16/0.7250
DRCN [45]		1775 K	43.56 s	25.93/0.7237	24.50/0.6224	24.55/0.5830	21.90/0.5809	23.42/0.7313
MemNet [38]		678 K	14.67 s	26.16/0.7414	24.38/0.6199	24.58/0.5842	21.89/0.5825	23.56/0.7387
MLWAN (ours)		1230 K	3.14 s	**26.89/0.7732**	**24.97/0.6574**	**24.81/0.5969**	**22.95/0.6371**	**24.51/0.7833**

**Table 2:** Comparisons on the number of network parameters, inference time, and PSNR/SSIM of different multi-scale SR methods.

Method	Scale	Params	Times	PSNR/SSIM			
				Set14	B100	Urban100	Manga109
MSSR [65]	2×	668 K	1.91 s	33.11/0.9133	31.94/0.8966	30.84/0.9149	-/-
LapSRN [23]		1307 K	0.40 s	32.99/0.9124	31.80/0.8952	30.41/0.9103	37.27/0.9740
MSWSR [43]		1228 K	2.66 s	33.23/0.9123	31.88/0.8929	31.14/0.9169	37.32/0.9733
MLWAN (ours)		1230 K	1.69 s	**33.47/0.9227**	**31.96/0.8974**	**31.34/0.9309**	**37.39/0.9733**
MSSR [65]	4×	668 K	1.91 s	28.05/0.7686	27.28/0.7256	25.19/0.7535	-/-
LapSRN [23]		1307 K	0.50 s	28.09/0.7700	27.32/0.7275	25.21/0.7562	29.09/0.8900
MSWSR [43]		1228 K	2.66 s	28.47/0.7776	27.48/0.7311	25.78/0.7744	30.01/0.8999
MLWAN (ours)		1230 K	2.70 s	**28.55/0.7797**	**27.67/0.7429**	**25.89/0.7779**	**30.16/0.9110**
MSWSR [43]	8×	1228 K	3.11 s	24.82/0.6338	24.74/0.5914	22.30/0.6045	24.21/0.7609
LapSRN [23]		1307 K	0.58 s	24.35/0.6200	24.54/0.5860	21.81/0.5810	23.39/0.7350
MLWAN (ours)		1230 K	3.14 s	**24.97/0.6574**	**24.81/0.5969**	**22.95/0.6371**	**24.51/0.7833**

**Table 3:** Comparisons on PSNR/SSIM of MLWAN with CSAM and without CSAM.

Method	Scale	PSNR/SSIM				
		Set5	Set14	B100	Urban100	Manga109
MLWAN	2×	37.68/0.9589	33.47/0.9227	31.96/0.8974	31.34/0.9309	37.39/0.9733
MLWAN without CSAM		37.48/0.9581	33.21/0.9119	31.81/0.8913	31.13/0.9174	37.35/0.9730
MLWAN	4×	32.19/0.8957	28.55/0.7797	27.67/0.7429	25.89/0.7779	30.16/0.9110
MLWAN without CSAM		32.02/0.8921	28.49/0.7782	27.50/0.7328	25.86/0.7774	30.07/0.9010
MLWAN	8×	26.89/0.7732	24.97/0.6574	24.81/0.5969	22.95/0.6371	24.51/0.7833
MLWAN without CSAM		26.76/0.7631	24.82/0.6339	24.74/0.5912	22.30/0.6044	24.22/0.7608

**Table 4:** Comparisons on PSNR/SSIM of MLWAN with ECA and without ECA.

Method	Scale	PSNR/SSIM				
		Set5	Set14	B100	Urban100	Manga109
MLWAN	2×	37.68/0.9589	33.47/0.9227	31.96/0.8974	31.34/0.9309	37.39/0.9733
MLWAN without ECA		37.31/0.9574	32.19/0.9123	31.88/0.8960	31.12/0.9169	37.31/0.9730
MLWAN	4×	32.19/0.8957	28.55/0.7797	27.67/0.7429	25.89/0.7779	30.16/0.9110
MLWAN without ECA		31.94/0.8910	28.41/0.7763	27.46/0.7310	25.70/0.7715	29.83/0.8974
MLWAN	8×	26.89/0.7732	24.97/0.6574	24.81/0.5969	22.95/0.6371	24.51/0.7833
MLWAN without ECA		26.71/0.7622	24.81/0.6338	24.75/0.5915	22.35/0.6063	24.23/0.7688

**Table 5:** Comparisons on PSNR/SSIM between MLWAN and LWCAN.

Method	Scale	PSNR/SSIM				
		Set5	Set14	B100	Urban100	Manga109
MLWAN	4×	32.19/0.8957	28.55/0.7797	27.67/0.7429	25.89/0.7779	30.16/0.9110
LWCAN		31.94/0.8910	28.41/0.7763	27.46/0.7310	25.70/0.7715	29.83/0.8974

## Data Availability

Not applicable.
